# Systematic review of clinical trials assessing the therapeutic efficacy of visceral leishmaniasis treatments: A first step to assess the feasibility of establishing an individual patient data sharing platform

**DOI:** 10.1371/journal.pntd.0005781

**Published:** 2017-09-05

**Authors:** Jacob T. Bush, Monique Wasunna, Fabiana Alves, Jorge Alvar, Piero L. Olliaro, Michael Otieno, Carol Hopkins Sibley, Nathalie Strub Wourgaft, Philippe J. Guerin

**Affiliations:** 1 Centre for Tropical Medicine and Global Health, University of Oxford, Oxford, United Kingdom; 2 Infectious Diseases Data Observatory (IDDO), Centre for Tropical Medicine and Global Health, Oxford, United Kingdom; 3 Drugs for Neglected Diseases initiative, Nairobi, Kenya; 4 Drugs for Neglected Diseases initiative, Geneva, Switzerland; 5 Special Programme on Research and Training in Tropical Diseases (WHO/TDR), Geneva, Switzerland; 6 Department of Genome Sciences, University of Washington, Seattle, Washington, United States of America; Saudi Ministry of Health, SAUDI ARABIA

## Abstract

**Background:**

There are an estimated 200,000 to 400,000 cases of visceral leishmaniasis (VL) annually. A variety of factors are taken into account when considering the best therapeutic options to cure a patient and reduce the risk of resistance, including geographical area, malnourishment and HIV coinfection. Pooled analyses combine data from many studies to answer specific scientific questions that cannot be answered with individual studies alone. However, the heterogeneity of study design, data collection, and analysis often makes direct comparison difficult. Individual Participant Data (IPD) files can be standardised and analysed, allowing detailed analysis of this merged larger pool, but only a small fraction of systematic reviews and meta-analyses currently employ pooled analysis of IPD. We conducted a systematic literature review to identify published studies and studies reported in clinical trial registries to assess the feasibility of developing a VL data sharing platform to facilitate an IPD-based analysis of clinical trial data. Studies conducted between 1983 to 2015 that reported treatment outcome were eligible.

**Principal findings:**

From the 2,271 documents screened, 145 published VL clinical trials were identified, with data from 26,986 patients. Methodologies varied for diagnosis and treatment outcomes, but overall the volume of data potentially available on different drugs and dose regimens identified hundreds or possibly thousands of patients per arm suitable for IPD pooled meta-analyses.

**Conclusions:**

A VL data sharing platform would provide an opportunity to maximise scientific use of available data to enable assessment of treatment efficacy, contribute to evidence-based clinical management and guide optimal prospective data collection.

## Author summary

Visceral leishmaniasis (VL) is a vector borne infectious disease caused by protozoan parasites transmitted by sand flies. The disease affects both immunocompetent and immunocompromised patients, predominantly occurring in remote regions in specific areas of Africa, Asia and South America. There are an estimated 200,000 to 400,000 cases each year, resulting in 20,000 to 40,000 deaths. Few therapeutic options are available, and these are typically expensive, not adapted for field use and cause life-threatening side effects. Treatment of the disease is further complicated by apparent variation in drug efficacy in patients from different regions. A comprehensive understanding of treatment outcomes is essential to ensure more effective treatment and control of VL. This systematic review was undertaken to determine whether sufficient relevant data are available to warrant creation of a data sharing platform for harmonisation and comparison of VL clinical trials. We identified 145 published VL clinical trials, which together have enrolled 26,986 patients. There was sufficient consistency in the trial methods to support pooling of these individual patient data. The findings of this review suggest that a VL data sharing platform would enable meaningful meta-analysis of VL treatment trials, which could inform treatment allocation, further drug development, and guide improved disease control.

## Introduction

Visceral Leishmaniasis (VL) is listed among the 18 neglected tropical diseases by the World Health Organization (WHO) [1,2]. VL is caused by protozoan parasites of the genus *Leishmania* and is transmitted by sand flies and is fatal if left untreated. WHO estimates that 200,000 to 400,000 cases occur each year (90% of which in six countries: India, Bangladesh, Sudan, South Sudan, Ethiopia and Brazil) with 20,000 to 40,000 deaths. There are uncertainties around these figures both because of underreporting, specially in the absence of field-adapted diagnostic tools, and because VL incidence in the Indian Subcontinent has dropped significantly as a result of the Kala-Azar Elimination Program (KEP) [3,4]. The disease affects the poorest of the poor, predominantly occurring in remote regions where there is limited access to healthcare [4]. There are few therapeutic options available and these are expensive, not field adapted, carry substantial toxicity, or a combination of these [5]. At present, the medicine development pipeline remains rather limited. There is a need for effective, safe, affordable and field adapted treatments for VL, as well as methods to detect the emergence of drug resistance [6,7].

Treatment of the disease is further complicated by large regional variation of drug efficacy due to either parasite or host factors, including high rates of relapse associated with HIV coinfection [8–10]. To improve the treatment of the disease, a comprehensive understanding of the factors affecting drug efficacy is essential, and current information could be mined from clinical trials already completed. Typically, summary statistics of trials are released, rather than individual patient data (IPD), and details of trial reports can lack standardisation and systematic reporting, making comparison of efficacy between drugs and regions challenging. Under these conditions, sophisticated meta-analysis that summarises the clinical trial landscape has not been achieved. In 2010 the World Health Organization (WHO) Expert Committee on the Control of Leishmaniases published a technical report *Control of the leishmaniases* [11], which highlighted the need for systematic reviews to summarise clinical trial results and improve consistency in trial design.

A vastly improved understanding of the clinical outcomes of VL treatment could be obtained through collection of IPD from clinical trials in a database. Such a database would provide a powerful tool to maximise the utility of the clinical data available and could serve two primary functions. First, it would provide an accessible, comprehensive and up-to-date archive of completed VL clinical trials, describing the protocols, methods and outcomes of each trial. This resource would be valuable to clinicians, drug developers and healthcare policy makers, helping to better inform the allocation and efficacy of VL treatments. The collation of all trials in one platform would also guide the design of future trials, improve consistency in trial parameters and could be used to promote international standards such as the Clinical Data Interchange Standards Consortium (CDISC) has developed for other diseases. Second, pooling of IPD would enable analyses of patient outcomes across trials. These analyses could provide a better understanding of the determinants of treatment efficacy and identify sub-populations of patients at particular risk of treatment failure due to factors such as age, geographic origin, or coinfection with other diseases.

The WorldWide Antimalarial Resistance Network (WWARN) has demonstrated the utility of an IPD repository for meta-analysis of clinical trials of antimalarial medicines, collecting IPD from over 400 trials involving more than 140,000 patients [12–18]. Furthermore, the network has developed methods and tools to harmonise, curate and pool IPD, integrating clinical, pharmacological, molecular, *in vitro* and other laboratory outputs to produce a platform enabling in-depth analysis of antimalarial treatment. Meta-analyses of these large standardised data sets have identified risks of treatment failure that were not detected in the much smaller original studies, or by classical aggregated meta-analyses.

Based on the malaria experience, the Infectious Diseases Data Observatory (IDDO) has been recently established and is building upon the methods and success developed by WWARN, but focused on neglected tropical diseases (NTDs) and emerging infections [19]. A similar approach based on IPD from VL clinical trials could identify similar risks to patients and help to overcome some of the challenges associated with the treatment of VL.

The first step in realising a VL data platform was to review comprehensively both published and unpublished sources to identify relevant studies on the efficacy and safety of VL drugs that could provide IPD for inclusion in the database. Initially, we evaluated the number of studies that could provide relevant data sets, to assess the potential size of such a database. Subsequently, information on the trial design, drug and dose regimen, geographical location, and patient characteristics was extracted to assess the extent to which meta-analysis could be conducted on pooled IPD. We report here the results of this comprehensive systematic review.

## Methods

### Literature search

This systematic review of studies on the efficacy of VL pharmaceuticals was conducted according to Preferred Reporting Items for Systematic Reviews and Meta-Analyses (PRISMA) guidance [20]. The following clinical trials registries and publication repositories were queried for all results published or registered before January 2016: clinicaltrials.gov, WHO International Clinical Trials Registry Platform (ICTRP), the Cochrane Library and PubMed. The last searches were performed between 26 January and 18 February 2016. The references in three VL reviews were also examined for relevant studies [6,21,22]. The search was divided into four stages:

1. Clinical trial registries clinicaltrials.gov and ICTRP were queried with the search terms ‘visceral leishmaniasis’ and ‘kala azar’.
2. PubMed was searched using the search term ‘Visceral Leishmaniasis’ and applying the ‘article type’ filter ‘clinical trial’.
3. A broader search of PubMed was conducted using the query ‘((kala AND azar) OR (visceral AND leishmaniasis)) AND (pentamidine OR ambisome OR amphotericin OR paromomycin OR miltefosine OR pentavalent OR sodium)’.
4. The Cochrane library was searched using the search term ‘Visceral Leishmaniasis’ and applying the ‘article type’ filter ‘clinical trial’.

### Eligibility screening

Publications and trials that described studies on cutaneous leishmaniasis, post kala-azar dermal leishmaniasis (PKDL), canine VL, vector control, nets, prevalence estimation, diagnostic tests, vaccines or prophylaxis were excluded. Non-intervention studies, case reports, retrospective studies and individual studies enrolling fewer than six patients were also excluded. There was no restriction placed on the date or language of the publications.

### Data extraction

Trial reports, publications and abstracts were manually examined and relevant information (Table 1) was recorded in a spreadsheet document (Microsoft Excel, available in the supplementary information, S1 Dataset). Fields for which information could not be found in a publication or online abstract were entered as “unknown” and were either excluded in the subsequent meta-analysis or included as “unknown”.

**Table 1:** Study parameters recorded in the spreadsheet.

Meta data	Study Characteristics	Patient information
Clinical trial ID Publication citation Author(s) information Location Start and end date Publication date Number of patients	Drug(s) and dose(s) Design (single arm, comparative, etc) Patient allocation (randomised, etc) Diagnostic method Initial test of cure (time and method) Final test of cure (time and method) Follow-up (duration and method) Inclusion/exclusion criteria: e.g. HIV status, if known	Age Gender

## Results

### Identification of relevant studies

**Fig 1:**
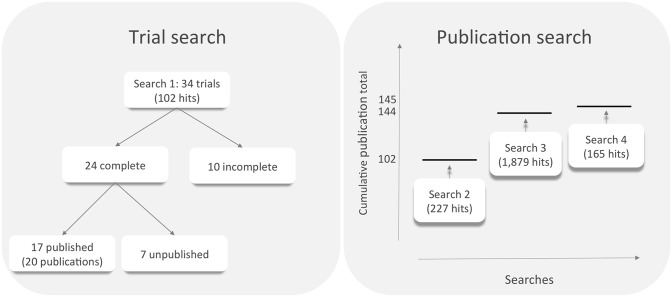
The number of visceral leishmaniasis clinical trials identified in searches of clinical trial registries and publication databases.

A query of clinical trials registries (clinicaltrials.gov and ICTRP) with the key words ‘visceral leishmaniasis’ and ‘kala azar’ returned 49 (clinicaltrials.gov) and 53 (ICTRP) search results, which contained 34 registered studies on the efficacy of VL drugs after filtering by exclusion criteria and removal of duplicates (Fig 1). These were annotated in the registries as complete (24), active (five), unknown (one), terminated (three) and withdrawn (one). Associated publications were found for 17 of the 24 completed trials. One of these trials was split into three studies and another was split into two studies, which were reported separately, giving 20 publications in total. The seven complete but unpublished trials were reported to have been initiated six to nine years ago, and aimed to enrol 1,746 patients. The active trials aim to enrol 1,804 patients in total.

It was anticipated that some trials might not have been registered with these registries; therefore, PubMed and the Cochrane library were queried for publications describing the results of relevant trials. Initially, PubMed was queried with the search term ‘visceral leishmaniasis’ and filtered for articles labelled with the tag ‘clinical trial’. This query returned 227 publications, of which 102 met the inclusion criteria (Fig 1). Subsequently, a broader search of PubMed was conducted using the query ‘*((kala AND azar) OR (visceral AND leishmaniasis)) AND (pentamidine OR ambisome OR amphotericin OR paromomycin OR miltefosine OR pentavalent OR sodium)*’. This search returned 1,879 results, of which 42 not previously identified studies met the inclusion criteria, bringing the total to 144 studies. In addition, the Cochrane library was queried with the search term ‘visceral leishmaniasis’ for articles associated with the label ‘clinical trial’, which added one relevant trial to the list, bringing the total to 145. This list included the 20 publications that were identified in the query of clinical trials registries. The reports were examined for duplication by comparison of the trial dates, dose regimes and trial sites, and none appeared to report overlapping patient data. Relevant parameters from these publications were manually extracted and entered into a spreadsheet (Table 1 and supplementary information, S1 Dataset).

### Characteristics of studies

**Fig 2:**
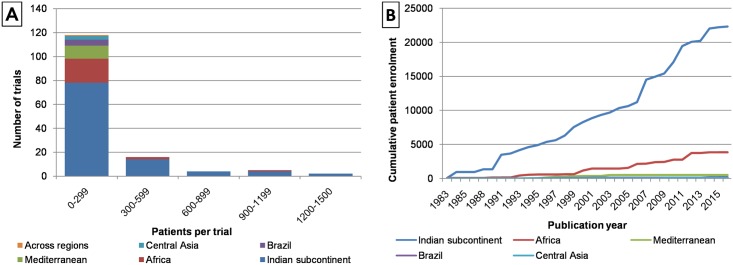
Size, location and date of visceral leishmaniasis clinical trials. **A**: The histogram shows the size distribution of visceral leishmaniasis clinical trials. Bars are coloured according to the region in which the trials were conducted. **B**: The cumulative enrolment of patients into clinical trials is shown as a function of the year in which the trial results were published. Data are divided according to the location at which the patients were enrolled.

The 145 VL clinical trials enrolled a total of 26,986 patients. The number of patients in individual trials ranged from 7 to 1,500 with a mean of 186 and a median of 85 patients per study (Table 2). One hundred and thirty-nine studies were conducted in a single country while the remaining six were multi-country studies. For the purpose of this analysis, patients were split into cohorts according to participating countries. A cohort was defined as a group of patients in a clinical trial who were enroled in a common country. The resulting 157 cohorts were distributed across five disease-relevant geographic regions: the Indian subcontinent (104 cohorts, 22,336 patients), Africa (30 cohorts, 3,836 patients), the Mediteranean basin (14 cohorts, 513 patients), Brazil (6 cohorts, 206 patients) and Central Asia (3 cohorts, 68 patients) (Fig 2A, Table 2). It is notable that the large majority of patients were enroled in the Indian sub-continent (66% cohorts, 83% patients). The number of patients enrolled in each of the five regions was plotted against the date of the reporting publication (Fig 2B). This graph indicates that clinical trials in the Indian subcontinent and Africa have been conducted regularly since 1990, and trials in other locations have been conducted since approximately 2000.

**Table 2:** The geographical distribution of studies and patients by country.

Country	Number of patients	Number of cohorts*
**Indian subcontinent**	**22336**	**104**
India	18901	95
Bangladesh	2670	4
Nepal	765	5
**Africa**	**3863**	**30**
Sudan	1675	10
Ethiopia	1280	6
Kenya	674	12
Uganda	234	2
**Mediterranean**	**513**	**14**
Italy	261	6
Greece	41	1
Spain	208	4
France	1**	1
Portugal	1**	1
Tunisia	1**	1
Brazil	206	6
**Central Asia**	**68**	**3**
Yemen	32	1
Iran	20	1
Saudi Arabia	16	1
**Grand Total**	**26986**	**157**

*Studies that were conducted in more than one country were split into cohorts, with one cohort for each of the participating countries.

** Clinical studies that enrolled <6 patients were excluded from this review. These cohorts containing <6 were part of multi-country studies.

### Patient characteristics

The majority of patients enrolled on the trials were male (16,122 male, 60%), 7,603 were female (28%), while the gender of the remaining 3,261 patients was not specified. The age threshold for inclusion varied between trials; 25 trials had a minimum age threshold for inclusion of 12 years or higher, and 14 trials set a maximum age threshold of 18 years or lower. The remaining 106 trials included both adult and child participants, or did not specify an age range.

The treatment of HIV coinfection was not clearly described in many publications. Six studies exclusively studied HIV coinfected patients, of which five were carried out in the Mediterranean basin and one in Africa. Eight trials tested for HIV upon enrolment and both HIV-negative and HIV-coinfected patients were included in the study. The majority of studies (131), however, did not test for HIV at enrolment. These trials either excluded patients with known HIV coinfection, tested for HIV only when a patient relapsed or made no mention of HIV.

### Drugs studied in visceral leishmaniasis clinical studies

**Fig 3:**
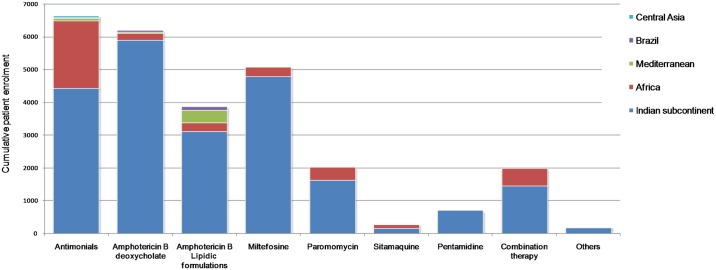
The number of patients treated with each drug in visceral leishmaniasis clinical studies. Bars are coloured according to the region in which the trials were conducted.

The majority of clinical studies investigated the efficacy of six drugs/drug-types: pentavalent antimonials (sodium stibogluconate and meglumine antimoniate; 6,638 patients), amphotericin B deoxycholate (6,191 patients), amphotericin B lipid-associated formulations (*e*.*g*. AmBisome, ABLC, Amphocil; 3,873 patients), miltefosine (5,067 patients), paromomycin (2,025 patients) and pentamidine (707 patients) (Fig 3). The three most studied drugs (pentavalent antimonials, amphotericin B deoxycholate and miltefosine) have each been used to treat more than 5,000 patients in clinical studies. Fifteen trials investigated combination therapies (1,988 patients). These were dominated by 10 studies on pentavalent antimonials, usually in combination with paromomycin, and three studies on liposomal amphotericin B in combination with miltefosine or paromomycin. Only 497 patients were treated with drugs outside these categories, of which 294 were treated with sitamaquine. Treatments with all of the six main drug types were mostly conducted in India. African patients were also represented for each drug type, however in smaller numbers, with the exception of pentavalent antimonials, which have been used to treat more than 2,000 patients in studies conducted in Africa.

**Fig 4:**
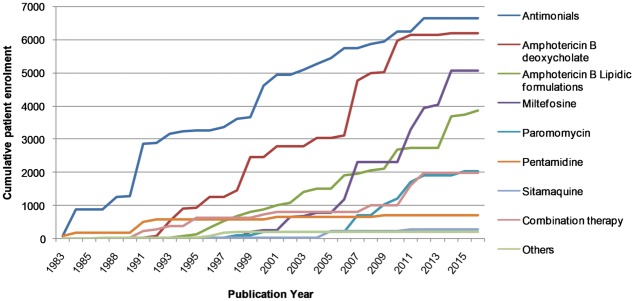
The temporal distribution of clinical trials on visceral leishmaniasis drugs. The cumulative enrolment of patients into clinical studies of VL drugs is shown as a function of the year in which the trial results were published.

The time periods during which these six major drug types have been studied were investigated by plotting the publication date of the trials against the cumulative number of patients enrolled (Fig 4). Trials of patients treated with pentavalent antimonials began in 1983, those testing amphotericin B deoxycholate in 1991, and trials of amphotericin B lipidic formulations in 1994. Trials of newer drugs, miltefosine, paromomycin or pentamidine have been published since 2000. Some trials of drug combinations have been conducted, primarily using lipidic amphotericin B or pentavalent antimonials in combination with other therapies.

### Dose regimens used in visceral leishmaniasis clinical studies

**Fig 5:**
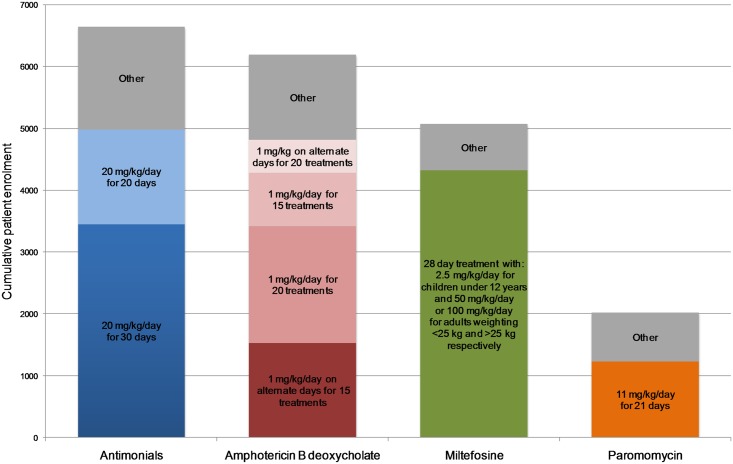
Bar chart showing dose regimens used in visceral leishmaniasis clinical studies. Bars show the number of patients treated with the drug in visceral leishmaniasis clinical studies. Bars are colored according to the dose regimen.

The consistency of dose regimens was assessed by analysing the doses used for the four most studied drugs (Fig 5, Table 3). Of the 6,638 patients treated with pentavalent antimonials, 4,981 received 20 mg/kg/day for either 20 days (1,532 patients) or 28–30 days (3,449 patients, for the purpose of this analysis 28 and 30 day regimens were grouped in one category). It is noteworthy that only one trial has directly compared the 20 and 30 day dose regimes, and that study enrolled just 27 patients per arm [23,24]. A total of 6,191 patients were treated with amphotericin B deoxycholate, of whom 4,808 received one of four dose regimes: 1 mg/kg/day for 20 consecutive days (1,890 patients), 20 alternate days (530 patients), 15 consecutive days (859 patients) or 15 alternate days (1,529 patients). The majority of trials using miltefosine (4,317 out of 5,067 patients) used the standard dose (28 day treatment with 2.5 mg/kg/day for children under 12 years, otherwise 50 mg/day was used if the patient was <25 kg and 100 mg/day if >25 kg). Finally, approximately one half of paromomycin treatments used a dose regimen of 11 mg/kg/day for 21 days (1,227 out of 2,025 patients).

**Table 3:** The drugs and dose regimens used in visceral leishmaniasis clinical studies.

Drug	Dose regimen	Number of patients	Total
Pentavalent antimonial	20 mg/kg/d, 20 days 20 mg/kg/d, 28–30 days Other	1,532 3,449 1,657	6,638
Amphotericin B deoxycholate	1 mg/kg/d, 20 doses 1 mg/kg/alternate days, 20 doses 1 mg/kg/d, 15 doses 1 mg/kg/alternate days, 15 doses Other	1,890 530 859 1,529 1,383	6,191
Miltefosine	Standard dose* Other	4,317 750	5,067
Amphotericin B lipid-associated formulations	Not analysed		3,873
Paromomycin	11 mg/kg/day Other	1,227 798	2,025
Combination therapies	Not analysed	-	1,988
Pentamidine	Not analysed	-	707
Sitamaquine	Not analysed	-	294
Other	Not analysed	-	203

*28 day treatment with 2.5 mg/kg/day for children under 12 years, otherwise 50 mg/day if < 25 kg and 100 mg/day if > 25 kg.

### Methods used in visceral leishmaniasis clinical studies: Patient allocation, diagnostics and follow-up

**Fig 6:**
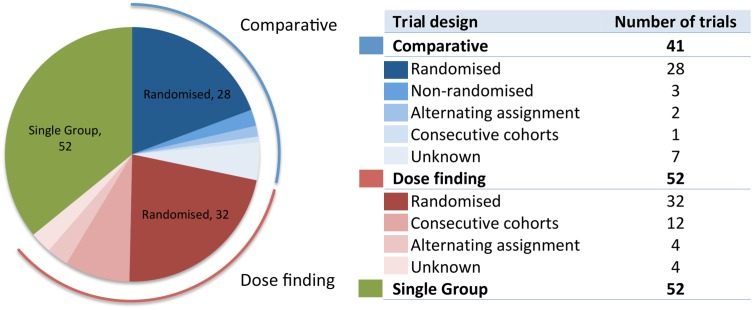
Pie chart and table showing the design and method of patient allocation in published visceral leishmaniasis clinical studies.

The studies were divided among dose-finding (52 studies), single-group assignments (52 studies) and comparative approaches (41 studies) (Fig 6). Only two trials were double-blinded and all others were either open-label or did not specify the method used. Randomised allocation of patients was used in the majority of the comparative (68%) and dose-finding (59%) trials. Consecutive cohorts of increasing or decreasing dose were also commonly employed in dose finding trials (23%).

**Fig 7:**
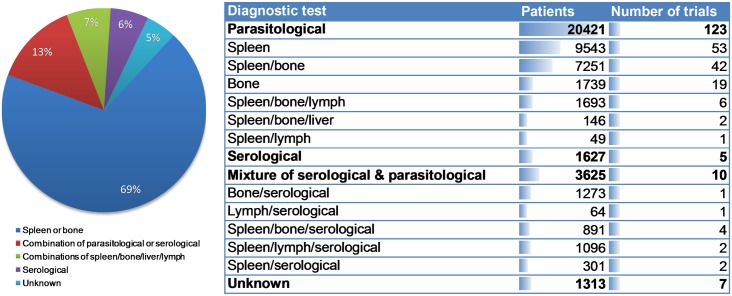
Pie chart and table showing diagnostic methods used in published visceral leishmaniasis clinical studies and the number of patients diagnosed by each method.

**Fig 8:**
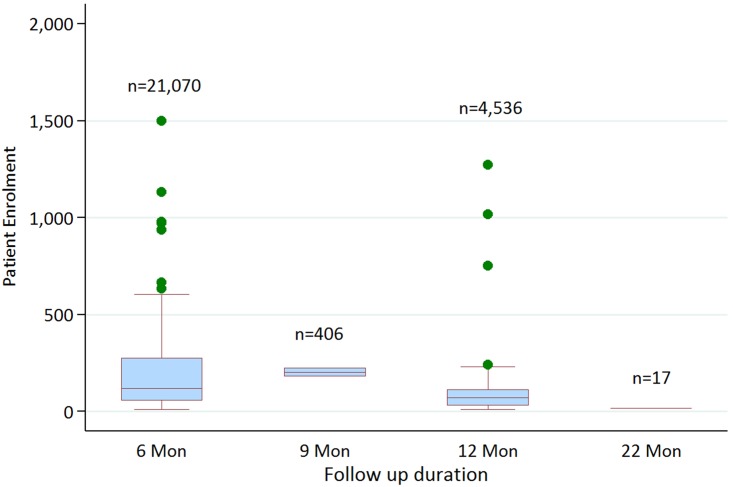
Box plot showing duration of follow-up in VL studies. The majority of studies (104 studies, 21,070 patients) had a follow-up duration of six months. Two studies (406 patients) had a nine month follow-up duration and 24 studies (4,546 patients) had a one year follow-up duration.

The consistency of the patient data collected was assessed by analysing the methods used for diagnosis, test of cure, and follow-up. The most common diagnostic test for VL was by microscopic observation of amastigotes in aspirates taken from the spleen or bone marrow (114 studies in total, 18,533 patients; spleen only (53 studies), bone marrow only (19 studies) or a mixture of the two (42 studies)). Nine studies used other combinations of aspirates, which included spleen, bone marrow, lymph nodes or liver. Serological only diagnoses were used in five trials, 10 trials used a mixture of serological and parasitological tests, and seven studies did not report the method of diagnosis (Fig 7). Almost all trials performed a parasitological test of cure within one month of end of treatment (120 trials, 20,680 patients), which was typically conducted using the same method as was used for diagnosis. The remaining 19 trials tested for cure either by a later parasitological test or assessment of clinical symptoms (seven studies did not provide this information). Patients were typically followed-up by monitoring for relapse of clinical symptoms, with a parasitological test only performed during the follow-up period in cases of apparent clinical relapse (102 studies). However, 37 studies performed a parasitological test at the end of follow-up for all enrolled patients (the remaining six studies did not provide this information). The length of follow-up was six months in almost all cases (21,070 patients), but in some studies it was 12 months (4,546 patients) (Fig 8).

### Delay to publication of the trials

The intervals between the reported completion of each trial and its publication date were calculated to obtain a measure of the delay before the trial outcomes were publically available. Trial end dates were reported in 87 of the publications and for these, the time from completion to publication was found to range from 8 to 80 months (6.7 years) with a mean delay of 28 months (median of 24 months). Futhermore, publications could not be found for seven of the studies that were annotated as complete in the clinical trials registries.

## Discussion

This review was undertaken to determine whether the size and consistency of existing VL clinical trial data is sufficient to warrant the construction of a pooled, harmonised database to enable meta-analysis of treatment outcomes. In particular, the number of patients studied, the similarity of the protocols, the location of the trials and the drugs tested were assessed. A systematic search of the literature identified 145 clinical trials, which enrolled more than 25,000 patients. There was some consistency in the trial design of most studies, allowing for a reasonable level of comparability if data were harmonised. The range of drugs, doses, participants, locations and methods used demonstrate the potential for clinically relevant meta-analyses and could guide prospective data collection.

This review identified a number of important issues regarding the methods used for reporting and curating trials. First, only 37 of the 162 completed studies identified here (both published and un-published) had been registered in either clinicaltrials.gov or the ICTRP. While it is true that some of these trials were conducted before the launch of clinicaltrials.gov in the year 2000, since then 74 studies have been published, of which only 20 were registered. The prevalence of un-registered trials highlights that retrieval and comprehensive analysis of VL clinical data will require substantial effort. The review also identified seven registered and completed clinical trials that did not have associated publications. Four of these studies were annotated in the clinical trials registries as being completed two to seven years ago; while the remaining three studies did not specify completion date in the registry. It is unclear whether the results of these studies will ultimately be published, and if not, these data could be lost. This finding is consistent with a study conducted by Riveros et al., which found that many registered trials in clinicaltrials.gov do not have associated publications [25]. A final issue with the current reporting of clinical trial results is the delay in publication. The review found that there was a two year delay to publication on average, and more than six years for some studies. These delays present an obstacle to timely access to important trial results, which could guide further research and policy.

An IPD repository could offer a solution to meet some of these issues. The database could employ semi-automatic upload and curation to actively capture newly registered clinical trials, rather than rely on registration by trial coordinators, thus ensuring that more trials are documented in a central repository. The database also could provide a platform for early sharing of clinical trial data, circumventing the delay between manuscript preparation / publication and meta-analyses. Additionally, data from trials that are not deemed suitable for publication in manuscript format could be deposited, ensuring these data are still available for inclusion in pooled analyses. Indeed, the platform could provide scientific support to speed publication of data.

One of the challenges in building this database will be locating the data from the 145 trials identified here. It has been reported that research data become increasingly difficult to locate with increasing age, for many reasons including hardware/software issues and lack of systematic storage processes [26]. It is therefore significant that most of the studies examined in this work (accounting for over 18,000 patients) were published after 2000, offering promise that much of the IPD will be accessible.

An effective database of IPD requires consistency in the methods used for diagnosis, test of cure and follow-up, to enable population of standardised data-fields. This review identified three key data-points that were collected in the majority of trials: (i) diagnosis was confirmed by detection of amastigotes in an aspirate for at least 76% of patients (>20,421 patients); (ii) a parasitological test of cure was performed within one month of completion of treatment for 87% of patients (23,507 patients); and (iii) nearly all patients were followed-up for at least six months (26,029 patients, 96%) either for clinical (20,706 patients) or parasitological (5,323 patients) relapse. Patients who were followed-up for clinical relapse typically received a parasitological test if clinical symptoms presented. In addition to good overall methodological consistency, there was sufficient variation in the methods (e.g. the aspirate used for parasitology or the length of follow-up), to suggest meta-analyses could inform decisions on the optimal design of trials and the selection of analytical methods.

Clinical trials have suggested there is substantial regional variation in the efficacy of VL drugs, which could be due to geographical variations in parasite response to the drugs and/or patient characteristics [9,27]. Additionally, there is concern that resistance may emerge to VL drugs, and therefore careful monitoring of their efficacy is required. Indeed, trials have found the efficacy of pentavalent antimonials in the Indian subcontinent has fallen over time, possibly because the drug had been deployed at low doses, with incremental increases with decreasing efficacy [24,28,29]. It is anticipated that an IPD repository could enable informative geospatial and temporal comparisons to determine the basis for apparent variations in drug efficacy, and facilitate monitoring for the emergence of drug resistance. The VL clinical studies identified here were carried out in a range of geographical areas and spanned almost three decades, suggesting meta-analyses across these datasets could be informative. While this systematic review excluded studies on PKDL, due to the large heterogeneity in the way the therapeutic outcomes are presented in the manuscripts, a VL IPD database could also investigate the potential for inclusion of data from PKDL studies.

Furthermore, due to the small scale of most VL clinical trials, few studies enrol sufficient patients of a specific age or gender to allow assessment of drug efficacy within subpopulations. Pooling data from many trials could provide the statistical weight to overcome these limitations, and so better inform treatment in the clinic. Finally, in addition to IPD, it would be advantageous to pool parasitological, biochemical and other laboratory data, to enable further analyses of outcome indicators.

### Conclusion

This systematic review identified a large number of studies on the efficacy of VL drugs, which together have enrolled more than 25,000 VL patients. The quantity, methodological consistency and diversity of the studies suggests that a centralised database of individual patient data would provide an effective tool for pooled analysis of treatment efficacy. Such analyses could investigate outcome variations associated with therapeutic interventions, regional influences, protocol variations and specific patient characteristics. This improved understanding of the determinants of treatment efficacy could provide a useful resource to policy makers and clinicians in allocating the most effective treatments for VL patients.

The data from this review are freely available from the IDDO website at iddo.org/vlsurveyor. Investigators interested to know more about the establishment of a VL data platform will find information at iddo.org/vl.

## Supporting information

S1 Dataset Spreadsheet of Visceral Leishmaniasis clinical trials and trial parameters. (XLSX)

S1 Prisma 2009 Flow Diagram (DOC)

S1 Prisma 2009 Checklist (DOC)
