# Supplementary material for: Systematic review of clinical trials assessing the therapeutic efficacy of visceral leishmaniasis treatments: A first step to assess the feasibility of establishing an individual patient data sharing platform
Source: PLoS Negl Trop Dis. 2017 Sep 5;11(9):e0005781. doi: 10.1371/journal.pntd.0005781 (PMC5600407; doi:10.1371/journal.pntd.0005781)
Supplement: S1 Prisma 2009 Flow Diagram — (DOC) [file pntd.0005781.s002.doc]

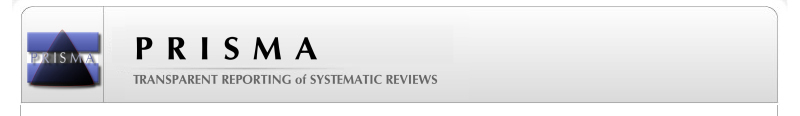
**PRISMA 2009 Flow Diagram**

**Screening**

**Included**

**Eligibility**

**Identification**

Records identified through database searching: pubmed (1,879), chocrane (180), clinicaltrials.gov (49) and ICTRP (53)
(n = 2,161)

Records screened based on title and abstract
(n = 2,161)

Records excluded
(n = 1,967)

Full-text articles assessed for eligibility
(n = 194)

Full-text articles excluded

(n = 49)

Exclusion criterea: studies on cutaneous leishmaniasis, [post kala-azar dermal leishmaniasis (](http://www.ncbi.nlm.nih.gov/pubmed/12560194)PKDL), canine VL, vector control, nets, prevalence estimation, diagnostic tests, vaccines or prophylaxis, non-intervention studies, case reports, retrospective studies and individual studies enrolling <6 patients

Studies included in qualitative synthesis
(n = 145)
